# Computed Tomography Scan and ICD Interaction

**DOI:** 10.1155/2009/189429

**Published:** 2009-11-01

**Authors:** Jose M. Porres, Jose L. Cerezuela, Oscar Luque, Pilar Marco

**Affiliations:** ^1^Arrhythmia Unit, Critical Care Department, Hospital Donostia, 20014 San Sebastian, Spain; ^2^CRM division, Boston Scientific Ibérica, Ribera del Loira 36, 28042 Madrid, Spain

## Abstract

Although it has been considered a safe procedure, computed tomography scanning uses high doses of radiation and can cause malfunctioning in those patients with ICD when the radiation is directly incident on the device. We present a case of ventricular oversensing during a thoracic computed tomography.

## 1. Text

A 65-year-old woman with a history of mixed connectivopathy (systemic scleroderma and lupus erythematosus) since 1984 and coronary artery disease with myocardial infarct since 1994 suffered a syncopal episode in March 2004.

During the electrophysiological study, a syncopal monomorphic ventricular tachycardia with a 240 milliseconds cycle length was induced. Ejection fraction was 45%.

On 12 March 2004 an ICD was implanted (Vitality 1871 DR; Boston Scientific, St Paul, MN, USA) with a right ventricular pace/sense and defibrillation lead (Endotak model 0148; Boston Scientific); values measured in at implant: capture threshold of 1.2 V at 0.5 milliseconds, measured *R* wave 20 mV, pacing impedance 614 Ohm and shocking impedance 48 Ohm, and right atrial pace/sense lead (1688 St Jude Medical, Sylmar, CA, USA).

Detection of VT was programmed at 180 beats/min, and VF at 220 beats/min. Ventricular sensitivity was programmed at 0.27 mV.

In January 2008 the elective replacement indicator (ERI) was reached and the device was replaced with Vitality 2 EL-T167 (Boston Scientific), maintaining the same programming. No ventricular arrhythmia had been detected by the device.

On 5 November 2008, as part of a study of pulmonary involvement by her rheumatic disorder, she underwent a high-resolution thoracic CT scan.

**Figure 1:**
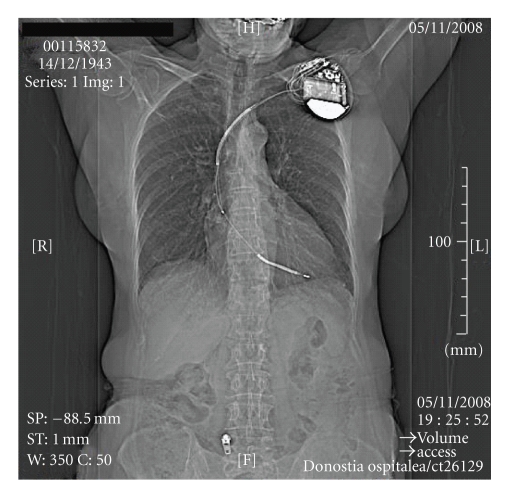
CT topogram. Initial topogram of the scan, showing the implanted device in the left pectoral zone and both electrodes. Arrows: date and time of scan.

This exam was conducted with a CT Volume Access Somaton (Siemens Medical Solutions, Forchheim, Germany). The study began at 19 : 25 and ended at 19 : 34; it yielded 36 images in 6 series together with the initial topogram (Figure 1). No complications occurred during the exam and the patient was sent home.

On 26 November 2008 the patient underwent ICD followup. All measurements of the battery and the leads remained within normal ranges; however, measurement of the intrinsic amplitude of the ventricle was impossible because the patient was pacing dependent.

**Figure 2:**
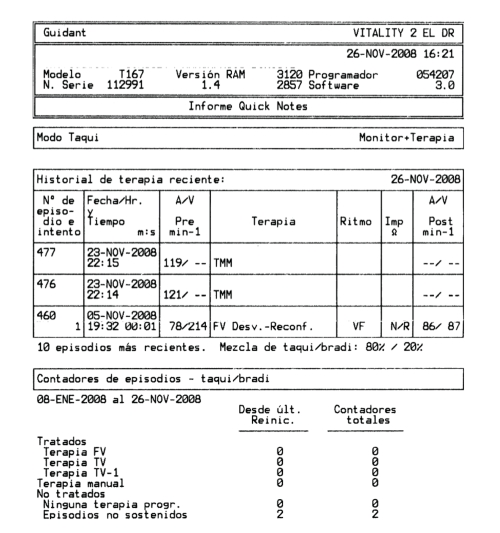
Therapy history: episode 460. Detection of ventricular fibrillation on November 5, 2008 at 19 : 32, coincident with the CT scan exam. Diverted therapy due to reconfirmation analysis.

The interrogation of the device-stored electrograms reveals an episode of nonsustained ventricular fibrillation which started capacitors charge that was diverted 3 seconds later (Figure 2).

**Figure 3:**
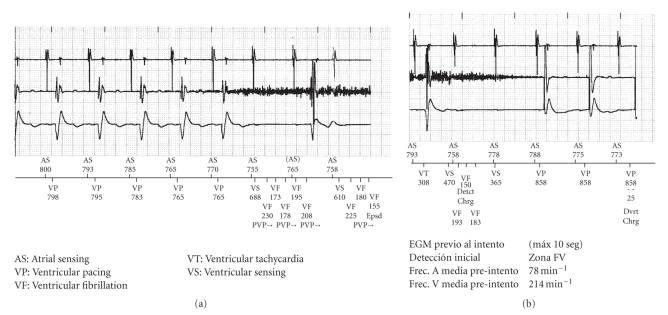
Electrograms of episode 460. Oversensing due to artifacts that start capacitors charge and provoke ventricular pacing inhibition.

Analysis of the stored electrograms of the episode, which coincided with the time of the thoracic CT scan, reveals an oversensing in the right ventricular channel interpreted by the device as ventricular activity. The oversensing provokes the charge of the capacitors and at the same time causes significant pauses due to inhibition of ventricular pacing (Figure 3). The patient did not show any symptoms during or after the exam.

Diaphragmatic or other usual causes of oversensing were excluded from the beginning by performing extreme breathing movements, left arm movements, and pocket manipulation in different patient positions.

Stability and integrity of both leads was confirmed with several followups during 6 months.

## 2. Discussion

The causes of interference affecting electronic cardiac devices are numerous and of diverse origin [1], but until only a few months ago CT was considered to be a safe procedure for patients with pacemakers and defibrillators. It was recommended, in a general way, as a substitute for magnetic resonance for those patients implanted with pacemakers or a defibrillator that required cross-sectional imaging.

CT did not appear in lists of potential sources of electromagnetic interference [2]. The first reference to the influence of CT on pacemakers [3] was subsequently confirmed by the same research team with a series of 11 patients with implanted Medtronic demand-type pacemaker, (Minneapolis, Minn, USA), in 6 of whom they detected a transitory malfunction through oversensing [4].

In order to confirm these results a prospective study was set up to analyse 13 pacemakers and 8 defibrillators, manufactured by Medtronic, subjected to ionizing radiation using CT systems with the production of images in spiral and dynamic mode at different dosage levels [5]. The devices were assessed on an anthropomorphic phantom with 3 cm of tissue-mimicking material in order to produce conditions similar to the clinical situation.

Oversensing was detected in the majority of the units, with inhibition of up to 4 seconds in some devices. It is not specified whether episodes of oversensing occur in the defibrillators that cause a diagnosis of tachycardia and the activation of a therapy.

A recent study [6] repeated this analysis on 33 pacemakers from 6 manufacturers and on 9 ICDs from 4 manufacturers, all affixed to a dummy.

Short episodes of oversensing were observed on 3 pacemakers, but none of the ICDs appeared to be affected. In addition, there were two curious observations. The units affected were those of most recent manufacture, and the 3 pacemakers that were affected remained undamaged during the repetition of the test when protected by a thin layer of copper.

Clinically implanted devices have shown the influence of CT on neurostimulators.

Three patients showed unexpected electrical stimulation when the radiation was directly incident on the device [7].

None of these reports show examples of electrograms with oversensing.

In July 2008, the [US]FDA collected all this information and issued a preliminary notification [8] that drew attention to the risk of effects on electronic devices including pacemakers, neurostimulators, and insulin pumps when patients undergo CT examination with direct exposure of the device to high doses of X-rays.

Both the FDA and ECRI Institute [7, 8] issue some recommendations regarding patients with implanted life-supporting electronic devices who have to undergo CT procedures directly over the device.

- Use the lowest possible dose of radiation and during the shortest possible time.
- Programme the device to “Off” (ICD) or in safety mode (pacemakers).
- Have a pacemaker/defibrillator programmer, an external defibrillator and emergency medication available.
- After the examination, reprogramme the device to “On” or back to the initial programming. Check the full and correct functioning of the device.

Due to the coincidence in time of both and the absence of other known interference causes, we can conclude that the oversensing is caused by the CT scan.

This is the first case demonstrating that an ICD implanted in a human subject may be affected during a CT scan; oversensing may occur, causing pacing inhibition and starting a VF episode detection with charge of the capacitors of the high-voltage circuit.

## 3. Conclusion

Although it has been considered a safe procedure, computed tomography scanning uses high doses of radiation and can cause malfunctioning in those patients with ICD when the radiation is directly incident on the device.

Radiologists need to be aware of this possibility so that they may avoid this radiation as far as possible and will have to be prepared to treat those patients if complications arise.

In addition, the patient should be examined by his/her doctor after the scan to check that the device has not been damaged.
